# Controlling the reactivity of La@C_82_ by reduction: reaction of the La@C_82_ anion with alkyl halide with high regioselectivity

**DOI:** 10.3762/bjoc.19.138

**Published:** 2023-12-11

**Authors:** Yutaka Maeda, Saeka Akita, Mitsuaki Suzuki, Michio Yamada, Takeshi Akasaka, Kaoru Kobayashi, Shigeru Nagase

**Affiliations:** 1 Department of Chemistry, Tokyo Gakugei University, Koganei, Tokyo 184-8501, Japan; 2 Department of Chemistry, Josai University, Sakado, Saitama 350-0295, Japan; 3 Tsukuba Advanced Research Alliance, University of Tsukuba, Ibaraki 305-8577, Japan; 4 Department of Theoretical Studies, Institute for Molecular Science, Okazaki 444-8585, Japan

**Keywords:** electron transfer, metallofullerene, radical, reduction

## Abstract

Endohedral metallofullerenes have excellent redox properties, which can be used to vary their reactivity to certain classes of molecules, such as alkyl halides. In this study, the thermal reaction of the La@*C*_2_*_v_*-C_82_ anion with benzyl bromide derivatives **1** at 110 °C afforded single-bonded adducts **2**–**5** with high regioselectivity. The products were characterized by matrix-assisted laser desorption/ionization time-of-flight mass spectrometry and visible–near infrared spectroscopy. The reaction of La@*C*_2_*_v_*-C_82_ with alkyl halides using the same conditions showed no consumption of La@*C*_2_*_v_*-C_82_, indicating that the reactivity of La@*C*_2_*_v_*-C_82_ toward alkyl halides was effectively increased by one-electron reduction. Single-crystal X-ray diffraction analysis of the single-bonded adduct **3a** revealed the addition site of the *p*-methoxybenzyl group on La@*C*_2_*_v_*-C_82_. Theoretical calculations indicated that the addition site carbons in neutral La@*C*_2_*_v_*-C_82_ have high spin density, whereas those in the La@*C*_2_*_v_*-C_82_ anion do not have high charge densities. Thus, the reaction is believed to occur via electron transfer, followed by the radical coupling of La@*C*_2_*_v_*-C_82_ and benzyl radicals, rather than by bimolecular nucleophilic substitution reaction of La@*C*_2_*_v_*-C_82_ anion with **1**.

## Introduction

Fullerenes, the third carbon allotrope, have unique spherical molecular structures and exhibit high reactivity as electron-deficient polyolefins. The excellent redox properties of fullerenes are useful for their chemical derivatization and practical applications [1–5]. Fullerene anions can be easily produced chemically or electrochemically. C_60_^2−^ is a strong electron donor and potential nucleophile that reacts with electrophiles [6–11]. The mechanism for the reaction of C_60_^2−^ with alkyl halides has been studied in detail by Fukuzumi et al., who found that the reaction occurs via electron transfer, followed by bimolecular nucleophilic substitution (S_N_2) reaction [8].

Endohedral metallofullerenes, wherein one or more metal atoms are encapsulated inside a fullerene cage, have garnered research interest [12–15]. The encapsulation of metal atoms can result in electron transfer from the metal atoms to the fullerene cage. Because of this intramolecular electron transfer, the characteristic properties of metallofullerenes, such as their redox potentials, are significantly different from those of empty fullerenes. For example, La@C_82_ has paramagnetic properties, and its formal electronic structure is described as La^3+^C_82_^3−^. We previously investigated the reaction of M@*C*_2_*_v_*-C_82_ ions (M = Y, La, Ce) with disilirane, which possesses high reactivity toward electron acceptors [16–17]. Interestingly, the reactivity of M@*C*_2_*_v_*-C_82_ toward disilirane was increased by the one-electron oxidation of M@*C*_2_*_v_*-C_82_. Moreover, the reaction was suppressed by the one-electron reduction of M@*C*_2_*_v_*-C_82_. These results suggest that oxidation and reduction reactions are useful for tuning the reactivity of metallofullerenes. Recently, remarkable reactivity of [M_3_N@*I_h_*-C_80_]^2−^ (M = Lu, Sc) toward benzal bromide was reported, demonstrating one possible reaction of the anion species of closed-shell endohedral metallofullerenes [18]. Although [Lu_3_N@*I_h_*-C_80_]^2−^ reacts with benzal bromide to afford a methanofullerene, [Sc_3_N@*I_h_*-C_80_]^2−^ did not react under the same conditions (*E*_ox_ [Lu_3_N@*I_h_*-C_80_]^2−^ = 1.80 V vs Fc^+^/Fc; *E*_ox_ [Sc_3_N@*I_h_*-C_80_]^2−^ = −1.62 V vs Fc^+^/Fc; C_60_^2−^ = −1.50 V vs Fc^+^/Fc)). The different reactivity of [M_3_N@*I_h_*-C_80_]^2−^ was explained by theoretical calculations. The charge density of the highest occupied molecular orbital (HOMO) was more highly localized on the fullerene cage for [Lu_3_N@*I_h_*-C_80_]^2−^, whereas it was more localized on the inside of the cluster for [Sc_3_N@*I_h_*-C_80_]^2−^.

A previous study reported that thermal treatment of La@*C*_2_*_v_*-C_82_ in the presence of 3-triphenylmethyl-5-oxazolidinone in toluene afforded four different benzylated La@*C*_2_*_v_*-C_82_ isomers [19]. Benzyl radicals may have been generated due to the involvement of azomethine ylide; however, the detailed mechanism has not been elucidated. In this article, we describe the thermal reaction of the La@*C*_2_*_v_*-C_82_ anion, activated by one-electron reduction, with benzyl bromide derivatives.

## Results and Discussion

**Scheme 1:**
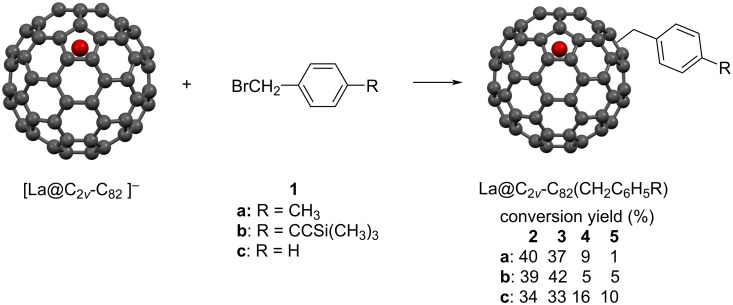
Reaction of the La@*C*_2_*_v_*-C_82_ anion with benzyl bromide derivatives.

**Figure 1:**
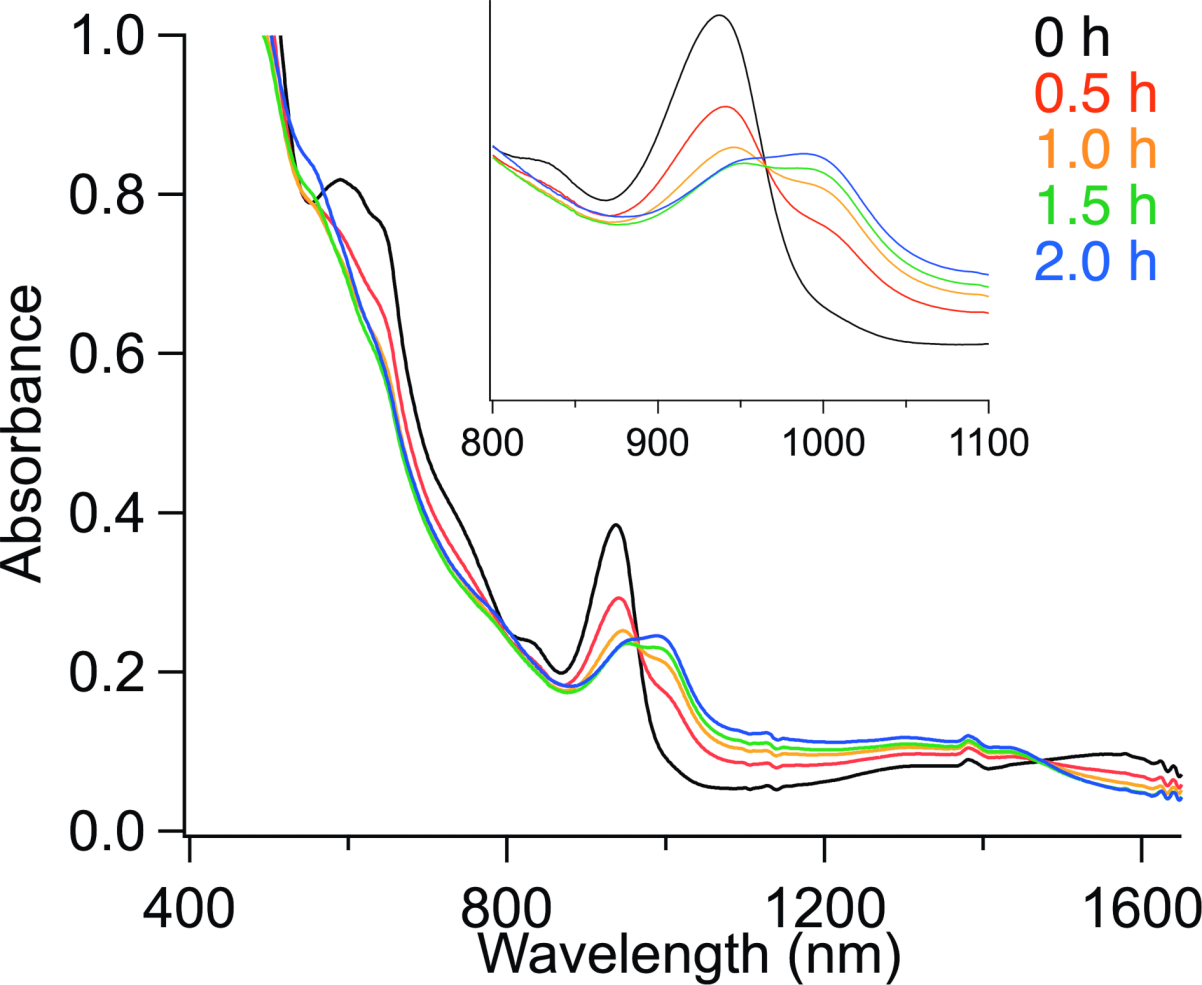
Changes in absorption spectra during the reaction of La@C_2_*_v_*-C_82_ anion with **1a**.

**Figure 2:**
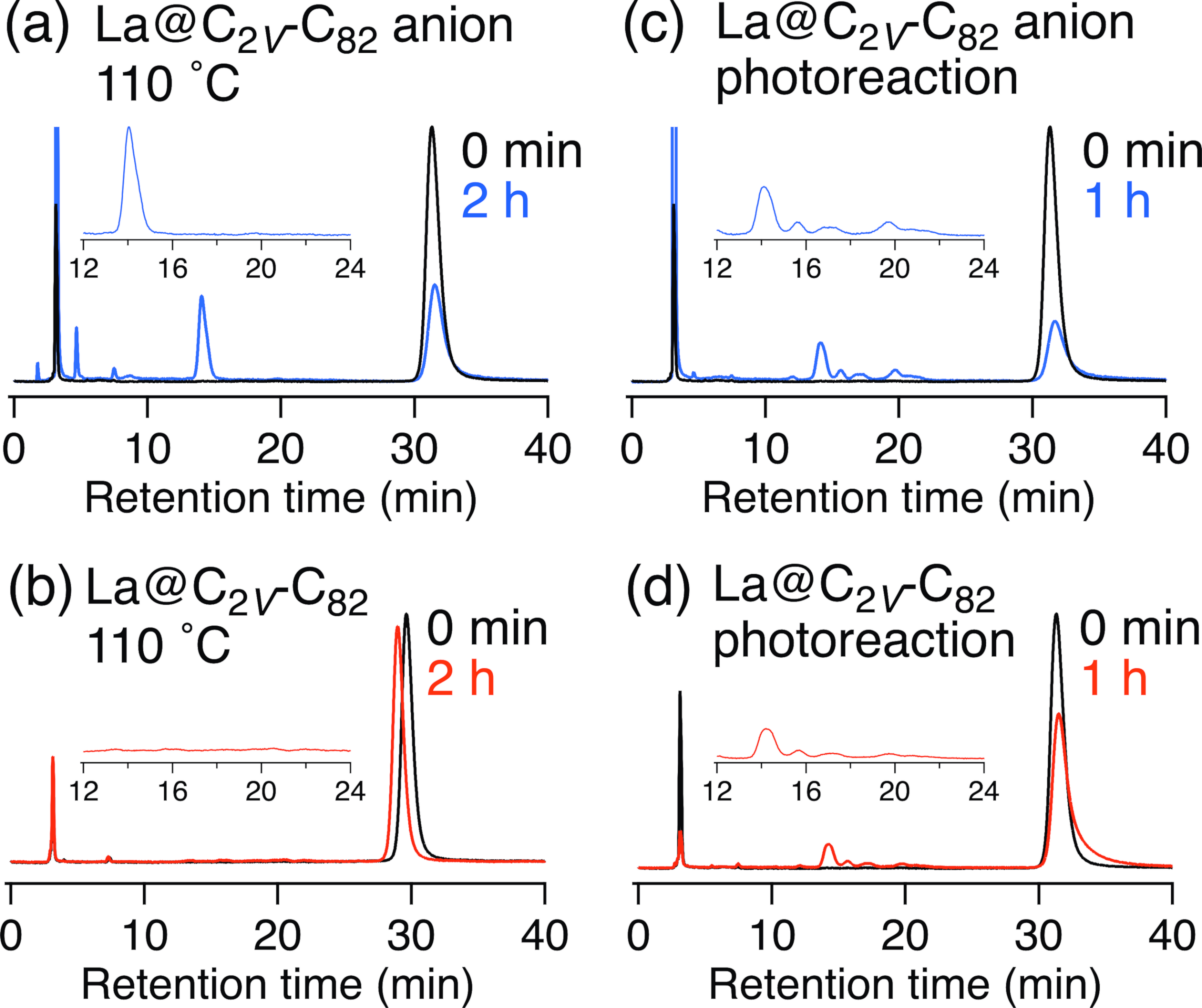
HPLC profiles of the reaction mixture. Conditions: Buckyprep column (⌀ = 4.6 × 250 mm); eluent, toluene; flow rate, 1 mL/min; UV detector, 330 nm. (a) La@*C*_2_*_v_*-C_82_ anion with **1a**. 110 °C, 2 h. (b) La@*C*_2_*_v_*-C_82_ with **1a**. 110 °C, 2 h. (c) La@*C*_2_*_v_*-C_82_ anion with **1a**. *hv* > 350 nm, 1 h. (d) La@*C*_2_*_v_*-C_82_ with **1a**. *hv* > 350 nm, 1 h. Black line is the HPLC profiles of La@C_2_*_V_*-C_82_ at the same concentration as the reaction. Reaction mixtures with La@*C*_2_*_v_*-C_82_ anions were treated with dichloroacetic acid before injection.

The La@*C*_2_*_v_*-C_82_ anion [20] was prepared by chemical reduction [21] using a degassed tetrabutylammonium hexafluorophosphate (TBAF) pyridine solution. After stirring for 3 h, a dark green solution was obtained. CS_2_ was added to precipitate TBAF, and the solution was filtered to collect the La@*C*_2_*_v_*-C_82_ anion solution. The solvent was then removed under reduced pressure and replaced with 1,2-dichlorobenzene (ODCB). The characteristic absorption peak at 1000 nm assigned to La@*C*_2_*_v_*-C_82_ decreased, and the new absorption peak at 934 nm assigned to the La@*C*_2_*_v_*-C_82_ anion increased. Reactions of the La@*C*_2_*_v_*-C_82_ anion with 4-methylbenzyl bromide (**1a**) were conducted at 110 °C for 2 h (Scheme 1). Figure 1 depicts the changes in the visible–near infrared (vis–NIR) absorption spectra during the reaction, showing gradual changes with isosbestic points. Since the electrolyte interferes with the high-performance liquid chromatography (HPLC) separation and anionic species may not be eluted under typical fullerene HPLC separation conditions, trifluoroacetic acid was added to the reaction mixture. Notably, La@*C*_2_*_v_*-C_82_ is produced after the addition of trifluoroacetic acid to the La@*C*_2_*_v_*-C_82_ anion [20]. After removing the solvent under vacuum, the electrolyte was removed by adding CS_2_ and then filtering. Subsequent HPLC separation of the reaction mixture with **1a** afforded products **2a**, **3a**, **4a**, and **5a** in yields of 40, 37, 9, and 1%, respectively, based on the consumption of La@*C*_2_*_v_*-C_82_ (Figure 2a and Supporting Information File 1, Figure S1). The yield was estimated from the absorption intensity ratio at 330 nm. On the other hand, no consumption of La@C*_2v_*-C_82_ was observed in the reaction of La@ C*_2v_*-C_82_ with **1a** (Figure 2b). The reaction of the La@*C*_2_*_v_*-C_82_ anion toward **2a**–**5a** requires heating, therefore the reactivity of La@*C_2v_*C_82_ anion is lower than that of the C_60_^2−^ and C_60_ anion radicals [10–11], which react even at room temperature. However, the one-electron reduction of La@*C*_2_*_v_*-C_82_ is effective for the activating its reactivity toward alkyl halides in the thermal reaction. Recently, Zhou et al. reported that the reaction of Gd@*C*_2_*_v_*-C_82_ with benzyl bromide requires a three-electron reduction of Gd@*C*_2_*_v_*-C_82_ for the addition reaction to occur at room temperature [22].

**Figure 3:**
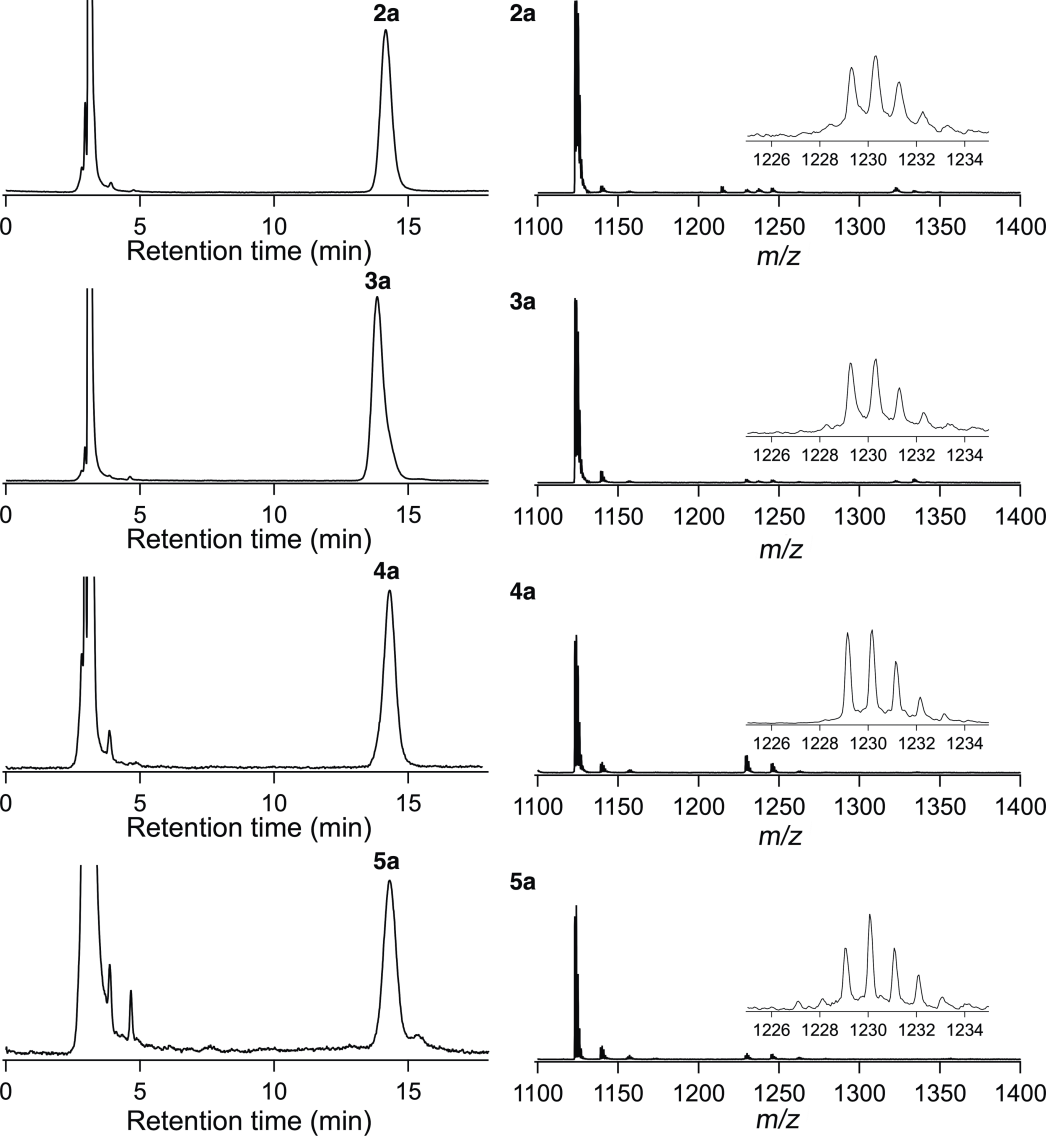
HPLC profiles (Buckyprep column (⌀ = 4.6 × 250 mm); eluent, toluene; flow rate, 1.0 mL/min; UV detector, 330 nm.) and MALDI–TOF mass spectra (matrix: dithranol, positive mode) of **2a**–**5a**.

Supporting Information File 1, Figure S1 depicts the three HPLC separation steps including recycling for the isolation. The matrix-assisted laser desorption/ionization time-of-flight (MALDI–TOF) mass spectra of **2a**–**5a** displayed the molecular ion peaks at *m*/*z* 1229, as expected for the 1:1 adducts of La@*C*_2_*_v_*-C_82_ and the 4-methylbenzyl group [MH]^+^ (Figure 3). Fragment peaks were observed at *m*/*z* 1123, corresponding to the mass of the fragment ion [La@*C*_2_*_v_*-C_82_]^+^. Similarly, the reaction of **1b** gave **2b**–**5b** in yields of 39, 42, 5, and 5%, respectively, and that of **1c** gave **2c**–**5c** in yields of 34, 33, 16, and 10%, respectively, based on the consumption of La@*C*_2_*_v_*-C_82_ (see Supporting Information File 1, Figures S2–S6).

For the comparison, the photoreaction of the La@*C*_2_*_v_*-C_82_ anion with **1a** was performed in ODCB using a high-pressure mercury arc lamp (cutoff < 350 nm, 1 h). The HPLC profile after the photoreaction indicates that several products other than **2a**–**5a** were present (Figure 2c), similar to the photoreaction of La@*C*_2_*_v_*-C_82_ with **1a** (Figure 2d). A previous study reported that the reaction of La@*C*_2_*_v_*-C_82_ with benzyl bromide under photolytic conditions affords eight monoadducts [19]. Therefore, one-electron reduction and the subsequent thermal reaction of La@*C*_2_*_v_*-C_82_ were effective for its functionalization in terms of both regioselectivity and reactivity compared to the thermal and photoreactions of La@*C*_2_*_v_*-C_82_ reported previously [19].

**Figure 4:**
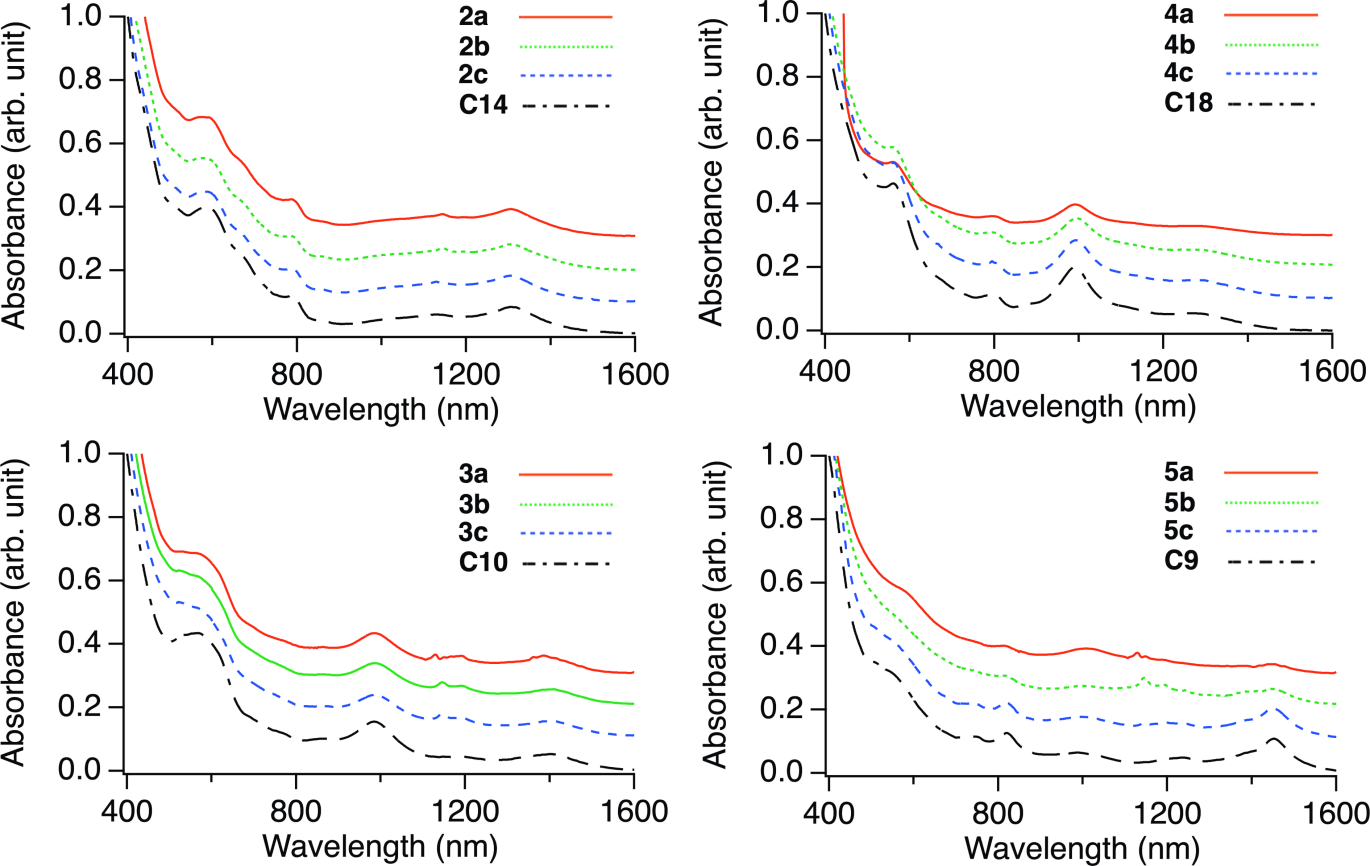
Absorption spectra of **2**, **3**, **4**, and **5** in CS_2_ and absorption spectra of C14, C10, C18, and C9 in Ce@*C*_2_*_v_*-C_82_(CH_2_C_6_H_3_Me_2_), which is attributed to the **1b**, **1d**, **1c**, and **1a** isomers in [25]. The carbon atom numbers correspond to that shown in Figure 6d.

Figure 4 shows the absorption spectra of **2**–**5**. Their absorption onsets move to shorter wavelengths relative to those of La@*C*_2_*_v_*-C_82_, which are characteristic features of single-bonded La@*C*_2_*_v_*-C_82_ derivatives [19,23], indicating that **2**–**5** have larger HOMO–lowest unoccupied molecular orbital energy gaps owing to their closed shell structures. As previous studies have shown that the absorption spectra of fullerene derivatives sensitively reflect the addition site, the absorption spectra can be regarded as powerful tools to determine the addition site in fullerene adducts [19,23–25]. Regardless of the substituents (**a**–**c**) of benzyl bromide, **2**, **3**, **4**, and **5** exhibited similar characteristic absorption features, respectively, supporting that the addition site of each isomer (e.g., **2a**–**c**) are the same.

**Figure 5:**
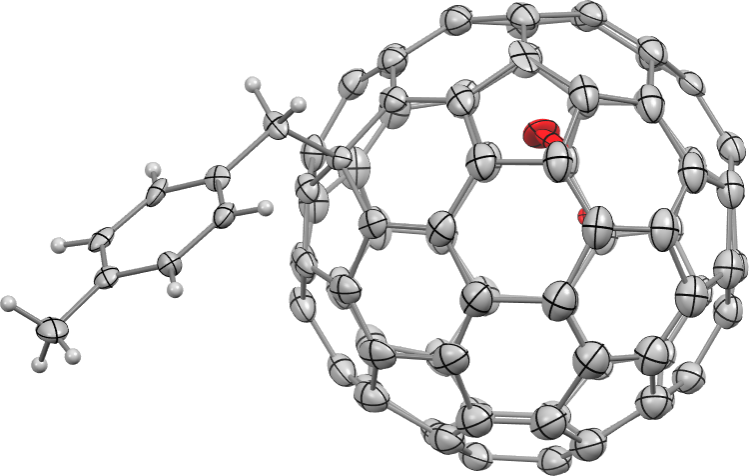
ORTEP drawing of **3a** with thermal ellipsoids shown at 50% probability level. Only an independent unit of **3a** is shown. This crystal has two independent units of **3a** and three CS_2_ molecules as guest solvents. The difference between the two independent units is the direction of the tolyl group in the crystal (Supporting Information File 1, Figure S7).

**Figure 6:**
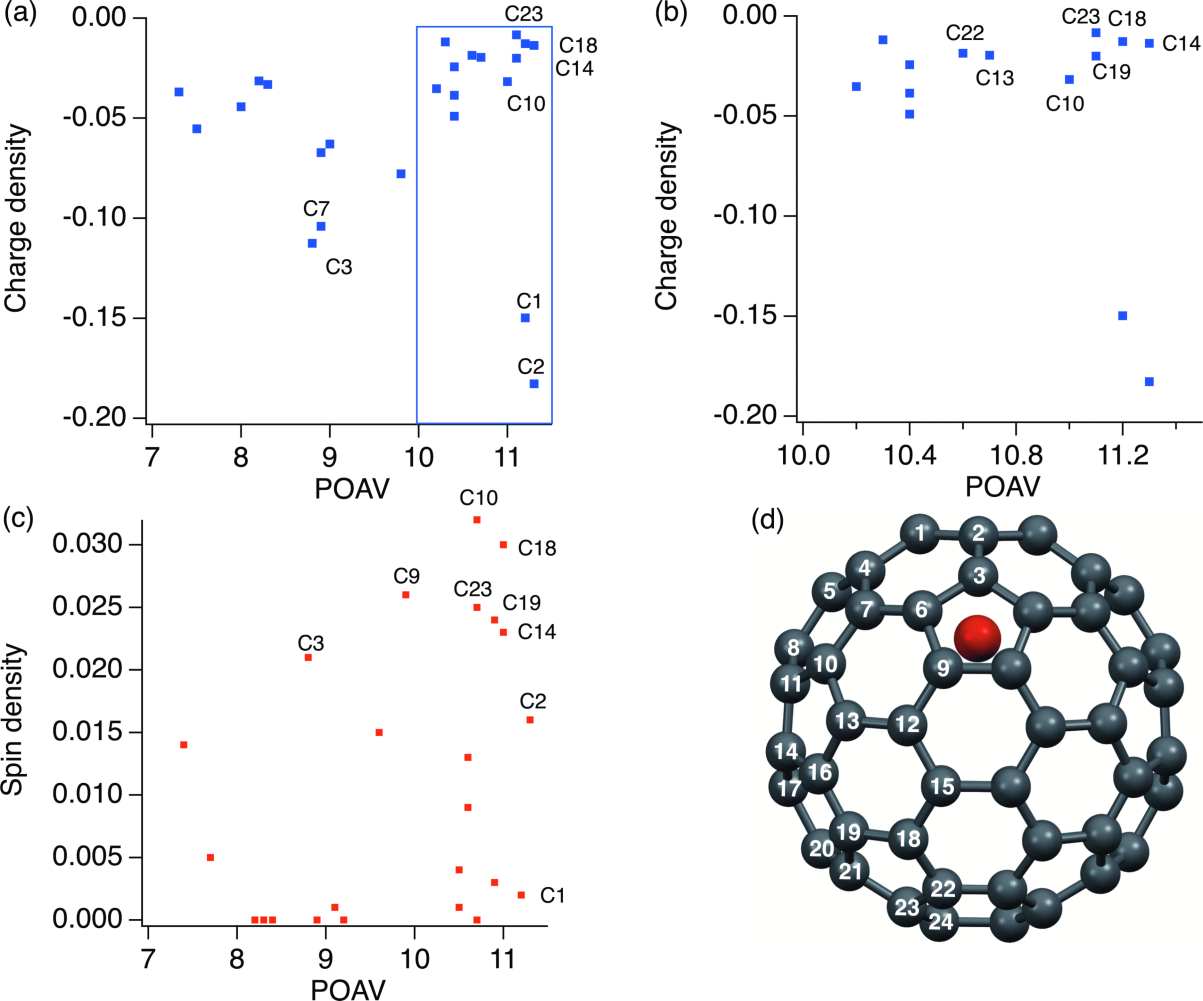
(a) Charge density of La@*C*_2_*_v_*-C_82_ anion as a function of its POAV values and (b) an enlarged part view of blue region in (a). (c) Spin density of La@*C*_2_*_v_*-C_82_ as a function of its POAV values [34–35]. (d) Molecular structure of La@*C*_2_*_v_*-C_82_ and numbering carbon atoms.

We determined the addition sites of the single-bonded La@*C*_2_*_v_*-C_82_ derivatives, La@*C*_2_*_v_*-C_82_(CHClC_6_H_3_Cl_2_) [19] and La@*C*_2_*_v_*-C_82_(CBr(CO_2_Et)_2_) [23], by single-crystal X-ray diffraction (SC-XRD) analysis. Based on the similarity in the absorption spectra of La@*C*_2_*_v_*-C_82_(CHClC_6_H_3_Cl_2_), the addition site of **3a**–**c** was expected to be at the C10 (for the numbering of carbon atoms in La@*C*_2_*_v_*-C_82_; see Figure 6d). Takano et al. estimated the addition sites of the 3,5-dimethylphenylmethyl group on Ce@*C*_2_*_v_*-C_82_ (Ce@*C*_2_*_v_*-C_82_(CH_2_C_6_H_3_Me_2_)) through temperature-dependent paramagnetic shifts of its nuclear magnetic resonance signals [25]. The similarity in the HPLC separation behavior and absorption spectra between the La@*C*_2_*_v_*-C_82_ adducts (**2a**–**c**, **3a**–**c**, and **4a**–**c**) [19] and the Ce@*C*_2_*_v_*-C_82_(CH_2_C_6_H_3_Me_2_) isomers [25] reported by Takano et al. was observed. Based on this observation, the plausible addition sites of **2a**–**c**, **3a**–**c**, and **4a**–**c** were estimated to be at the C14, C10, and C18 positions. The molecular structure of **3a** was confirmed by the SC-XRD analysis, which showed that the addition site of addendum was indeed at the C10 position of La@*C*_2_*_v_*-C_82_ (Figure 5).

The La@*C*_2_*_v_*-C_82_ anion can act as an electron donor and a nucleophile. To confirm the reaction mechanism, charge density and the p-orbital axis vector (POAV) values [26] of the carbon atoms (θ_σπ_-90˚) of the La@*C*_2_*_v_*-C_82_ anion were calculated using density functional theory (DFT) [27–33]. As shown in Table 1 and Figure 6, the C1, C2, and C3 atoms have large negative charge densities (C1: −0.1498, C2: −0.1828, C3: −0.1126), and C1 and C2 atoms have high POAV values (C1: 11.2, C2: 11.3) in the La@*C*_2_*_v_*-C_82_ anion. Meanwhile, the C10, C14, and C18 atoms have moderate or small negative charge densities (C10: −0.0317, C14: −0.0137, C18: −0.0128) and high POAV values (C10: 11.0, C14: 11.3, C18: 11.2) in the La@*C*_2_*_v_*-C_82_ anion. On the other hand, the C10, C14, and C18 atoms have larger spin densities (C10: 0.032, C14: 0.023, C18: 0.030) [34–35] than the C1 and C2 atoms (C1: 0.002, C2: 0.016) in La@*C*_2_*_v_*-C_82_ (Figure 6b). These results suggest that the reaction mechanism involving the electron transfer from the La@*C*_2_*_v_*-C_82_ anion to benzyl bromide derivatives followed by the radical coupling reaction is more plausible for the formation of the corresponding adducts rather than the S_N_2 reaction mechanism of the La@*C*_2_*_v_*-C_82_ anion with benzyl bromide derivatives.

**Table 1:** Charge densities and POAV values of carbon atoms for La@*C*_2_*_v_*-C_82_ anion, and spin densities and POAV values of carbon atoms for La@*C*_2_*_v_*-C_82_ [34–35]. The carbon atom numbers correspond to that shown in Figure 6d.

Carbon atom number	La@*C*_2_*_v_*-C_82_ anion		La@*C*_2_*_v_*-C_82_ [34–35]	
	POAV	Charge density	POAV	Spin density

1	11.2	−0.1498	11.2	0.002
2	11.3	−0.1828	11.3	0.016
3	8.8	−0.1126	8.8	0.021
4	9.8	−0.0778	9.6	0.015
5	9.0	−0.0629	9.2	0.000
6	10.4	−0.0491	10.5	0.004
7	8.9	−0.1041	9.1	0.001
8	8.9	−0.0672	8.9	0.000
9	10.2	−0.0353	9.9	0.026
10	11.0	−0.0317	10.7	0.032
11	10.4	−0.0386	10.6	0.009
12	7.5	−0.0554	7.7	0.005
13	10.7	−0.0196	10.9	0.003
14	11.3	−0.0137	11.0	0.023
15	7.3	−0.0369	7.4	0.014
16	10.4	−0.0244	10.6	0.013
17	8.0	−0.0443	8.2	0.000
18	11.2	−0.0128	11.0	0.030
19	11.1	−0.0201	10.9	0.024
20	8.3	−0.0332	8.4	0.000
21	10.3	−0.0119	10.5	0.001
22	10.6	−0.0186	10.7	0.000
23	11.1	−0.0084	10.7	0.025
24	8.2	−0.0314	8.3	0.000

## Conclusion

The reaction of La@*C*_2_*_v_*-C_82_ anion with benzyl bromide derivatives **1** at 110 °C afforded the corresponding single-bonded adducts **2**–**5** with high regioselectivity. One-electron reduction of La@*C*_2_*_v_*-C_82_ increased its reactivity during thermal reaction relative to that of neutral La@*C*_2_*_v_*-C_82_. Structural analysis of the two major products indicated that the characteristic absorption features were strongly affected by the addition sites. Based on theoretical studies and considering the identified addition sites, a plausible reaction mechanism for the reaction is the electron transfer from La@*C*_2_*_v_*-C_82_ anion to benzyl bromide, followed by radical coupling. This demonstrates that one-electron reduction of La@*C*_2_*_v_*-C_82_ is an easy and effective method for controlling its reactivity and selectivity via ionization for the production of La@*C*_2_*_v_*-C_82_ derivatives.

## Experimental

**General:** All chemicals and solvents were obtained from Wako, TCI, and Aldrich and were used without further purification unless otherwise stated. ODCB was distilled over P_2_O_5_ under vacuum prior to use. HPLC was performed on an LC-9201 instrument (Japan Analytical Industry Co., Ltd.) by monitoring the UV absorption at 330 nm with toluene as the eluent. Mass spectrometry was performed using a Bruker AUTOFLEX III smart beam with dithranol as the matrix. Optical absorption spectra were recorded using a Pyrex cell with a 10 mm path length and a spectrophotometer (V-670; Jasco Corp.).

### Preparation of La@*C*_2_*_v_*-C_82_

As described in [19], soot containing endohedral metallofullerenes were produced through the standard arc vaporization method using a composite anode rod containing graphite and metal oxide. The composite rod was subjected to an arc discharge under a He atmosphere at 50 Torr. Raw soot was collected and suspended in 1,2,4-trichlorobenzene (TCB). The mixture was refluxed for 16 h. The TCB solution was collected and injected into the HPLC instrument to separate the endohedral metallofullerenes using a PBB column (⌀ 20 mm × 250 mm; Cosmoses, Nacalai Tesque Inc.) with chlorobenzene as the mobile phase in the first step and a Buckyprep column (⌀ 20 mm × 250 mm × 2; Cosmoses, Nacalai Tesque Inc.) with toluene as the mobile phase in the second step.

### Preparation of the La@*C*_2_*_v_*-C_82_ anion

As described in [21], La@*C*_2_*_v_*-C_82_ (0.34 × 10^−6^ mol) was dissolved in 10 mL of a pyridine solution containing TBAF (0.54 × 10^−3^ mol) and then stirred for 2 h under an Ar atmosphere. The resulting green solution was concentrated to 2.0 mL. CS_2_ was added to the solution to precipitate excess TBAF which was then removed by filtration. The La@*C*_2_*_v_*-C_82_ anion ([La@*C*_2_*_v_*-C_82_]PF_6_) was collected as the filtrate (with a 78% yield estimated from the molar absorbance coefficient). This step was repeated to ensure a sufficient amount of La@*C*_2_*_v_*-C_82_ anions for the next step.

### Reaction of the La@*C*_2_*_v_*-C_82_ anion with **1a**

**1a** was added to 12 mL of the La@*C*_2_*_v_*-C_82_ anion (0.89 × 10^−6^ mol) ODCB solution. The solution was degassed using freeze-pump-thaw cycles. The solution was then heated at 110 °C for 2 h. After the reaction, dichloroacetic acid was added to recover the unreacted La@*C*_2_*_v_*-C_82_ in its neutral form. Four isomers, **2a**, **3a**, **4a**, and **5a**, and La@*C*_2_*_v_*-C_82_ were isolated from the reaction mixture using multistep HPLC, as shown in Supporting Information File 1, Figures S2, S4, and S6. The yields were calculated from the HPLC peak areas monitored at 330 nm, assuming that La@*C*_2_*_v_*-C_82_ and the monoadducts have the same absorption coefficients.

### X-ray crystallography

Black crystalline rods of **3a** were obtained using the liquid–liquid bilayer diffusion method with **3a** in a CS_2_ solution and an *n*-hexane solution in a glass tube (⌀ = 7 mm) at room temperature. The SC-XRD measurement was performed at 90 K on a Bruker AXS instrument equipped with an Apex II CCD detector with Mo *K*α radiation (λ = 0.71073 Å). The multi-scan method was used for absorption corrections. Structures were solved using direct methods and refined using SHELXL-2014/7 [36–38]. Deposition Number 2299232 (for **3a**) contains the supplementary crystallographic data for this study. This data was provided free of charge by the joint Cambridge Crystallographic Data Centre and Fachinformationszentrum Karlsruhe Access Structures Service.

### Theoretical calculations

POAV (θ_σπ_−90º) and charge densities values were calculated using the Gaussian 03 program with DFT at the B3LYP/3-21G for C and H [33], and the LanL2DZ basis set and effective core potential (ECP) for La [29,32].

**La@*C*_2_*_v_*-C_82_(CH_2_C_6_H_4_CH_3_)** (**2a**): vis–NIR (CS_2_): λ_max_ = 572, 741, 1304 nm; MALDI–TOF MS (*m*/*z*): [MH]^+^ calcd for LaC_90_H_9_, 1228.98; found, 1229.04.

**La@*C*_2_*_v_*-C_82_(CH_2_C_6_H_4_CH_3_)** (**3a**): vis–NIR (CS_2_): λ_max_ = 560, 851, 991, 1271 nm; MALDI–TOF MS (*m*/*z*): [MH]^+^ calcd for LaC_90_H_9_, 1228.98; found, 1228.99.

**La@*C*_2_*_v_*-C_82_(CH_2_C_6_H_4_CH_3_)** (**4a**): vis–NIR (CS_2_): λ_max_ = 525, 986, 1410 nm; MALDI–TOF MS (*m*/*z*): [MH]^+^ calcd for LaC_90_H_9_, 1228.98; found, 1229.15.

**La@*C*_2_*_v_*-C_82_(CH_2_C_6_H_4_CH_3_)** (**5a**): vis–NIR (CS_2_): λ_max_ = 809, 997, 1454 nm; MALDI–TOF MS (*m*/*z*): [MH]^+^ calcd for LaC_90_H_9_, 1228.98; found, 1229.04.

**La@*C*_2_*_v_*-C_82_(CH_2_C_6_H_4_CCSi(CH_3_)_3_)** (**2b**): vis–NIR (CS_2_): λ_max_ = 568, 762, 1307 nm; MALDI–TOF MS (*m*/*z*): [MH]^+^ calcd for LaC_94_H_16_Si, 1311.01; found, 1311.25.

**La@*C*_2_*_v_*-C_82_(CH_2_C_6_H_4_CCSi(CH_3_)_3_)** (**3b**): vis–NIR (CS_2_): λ_max_ = 559, 883, 995, 1242 nm; MALDI–TOF MS (*m*/*z*): [MH]^+^ calcd for LaC_94_H_16_Si, 1311.01; found, 1311.37.

**La@*C*_2_*_v_*-C_82_(CH_2_C_6_H_4_CCSi(CH_3_)_3_)** (**4b**): vis–NIR (CS_2_): λ_max_ = 521, 986, 1410 nm; MALDI–TOF MS (*m*/*z*): [MH]^+^ calcd for LaC_94_H_16_Si, 1311.01; found, 1311.29.

**La@*C*_2_*_v_*-C_82_(CH_2_C_6_H_4_CCSi(CH_3_)_3_)** (**5b**): vis–NIR (CS_2_): λ_max_ = 809, 998, 1447 nm; MALDI–TOF MS (*m*/*z*): [MH]^+^ calcd for LaC_94_H_16_Si, 1311.01; found, 1311.25.

**La@*C*_2_*_v_*-C_82_(CH_2_C_6_H_5_)** (**2c**): vis–NIR (CS_2_): λ_max_ = 583, 786, 1305 nm; MALDI–TOF MS (*m*/*z*): [MH]^+^ calcd for LaC_89_H_7_, 1214.97; found, 1214.83.

**La@*C*_2_*_v_*-C_82_(CH_2_C_6_H_5_)** (**3c**): vis–NIR (CS_2_): λ_max_ = 561, 891, 993, 1270 nm; MALDI–TOF MS (*m*/*z*): [MH]^+^ calcd for LaC_89_H_7_, 1214.97; found, 1214.96.

**La@*C*_2_*_v_*-C_82_(CH_2_C_6_H_5_)** (**4c**): vis–NIR (CS_2_): λ_max_ = 521, 986, 1410 nm; MALDI–TOF MS (*m*/*z*): [MH]^+^ calcd for LaC_89_H_7_, 1214.97; found, 1214.82.

**La@*C*_2_*_v_*-C_82_(CH_2_C_6_H_5_)** (**5c**): vis–NIR (CS_2_): λ_max_ = 818, 999, 1451 nm; MALDI–TOF MS (*m*/*z*): [MH]^+^ calcd for LaC_89_H_7_, 1214.97; found, 1214.98.

## Supporting Information

Supporting Information features HPLC chromatographs and MS spectra of fullerene derivatives, changes in absorption spectra during the reaction of La@*C*_2_*_v_*-C_82_ with **1b** and **1c**, X-ray crystallographic data of **3a**, and ORTEP drawings of the independent unit of **3a**.

File 1 Additional experimental data.
