# Supplementary material for: Controlling the reactivity of La@C82 by reduction: reaction of the La@C82 anion with alkyl halide with high regioselectivity
Source: Beilstein J Org Chem. 2023 Dec 11;19:1858–66. doi: 10.3762/bjoc.19.138 (PMC10729119; doi:10.3762/bjoc.19.138)
Supplement: File 1 — Additional experimental data. [file Beilstein_J_Org_Chem-19-1858-s001.pdf]

BEILSTEIN JOURNAL OF ORGANIC CHEMISTRY

# Supporting Information

for

# Controlling the reactivity of La@$C_{82}$ by reduction: reaction of the La@$C_{82}$ anion with alkyl halide with high regioselectivity

Yutaka Maeda, Saeka Akita, Mitsuaki Suzuki, Michio Yamada, Takeshi Akasaka, Kaoru Kobayashi and Shigeru Nagase

*Beilstein J. Org. Chem.* **2023,** *19,* 1858–1866. doi:10.3762/bjoc.19.138

# Additional experimental data

License and Terms: This is a supporting information file under the terms of the Creative Commons Attribution License (https://creativecommons.org/licenses/by/4.0). Please note that the reuse, redistribution and reproduction in particular requires that the author(s) and source are credited and that individual graphics may be subject to special legal provisions.
The license is subject to the *Beilstein Journal of Organic Chemistry* terms and conditions: (https://www.beilstein-journals.org/bjoc/terms)

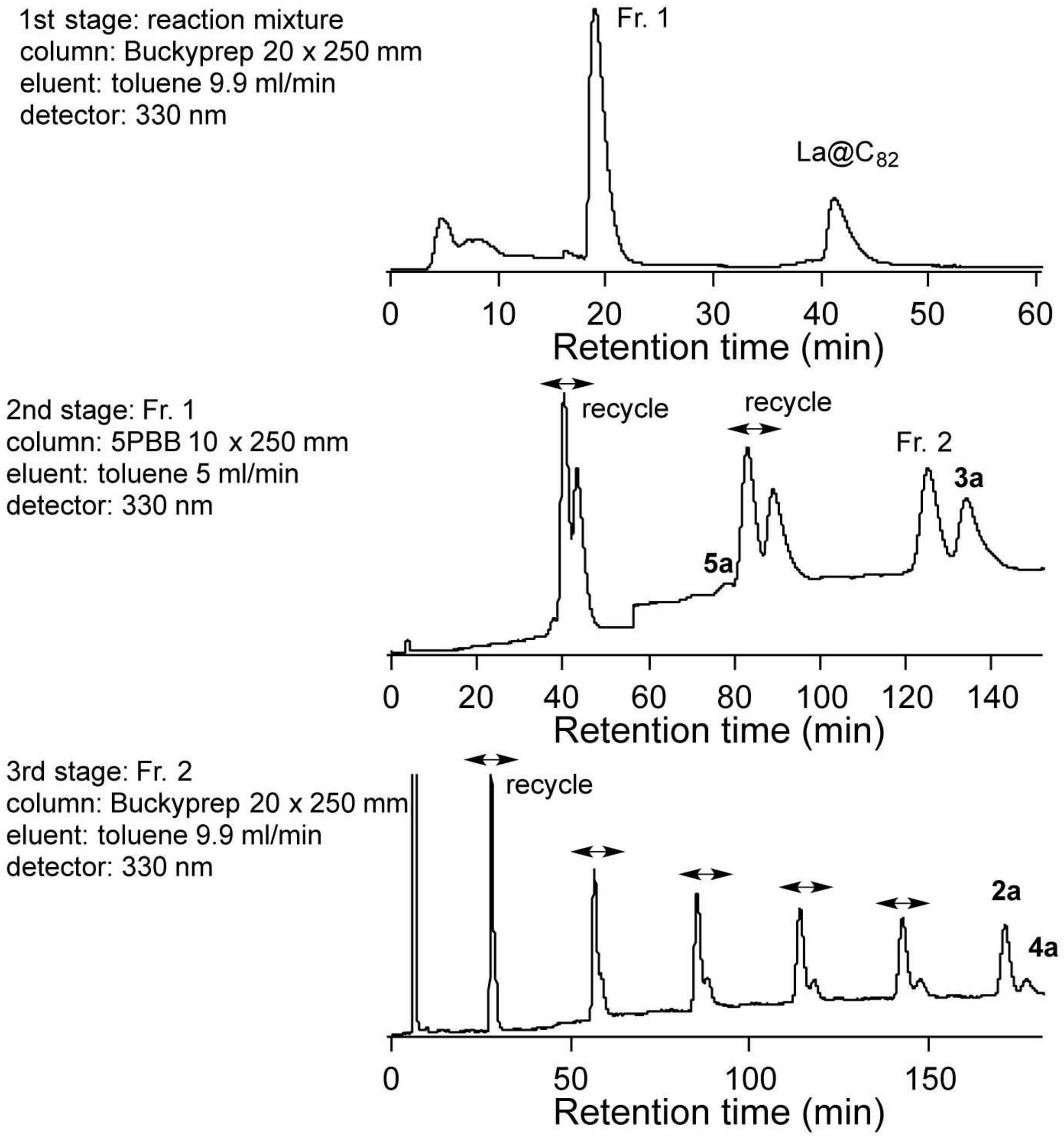


**Figure S1:** HPLC separation/isolation schemes for **2a**, **3a**, **4a**, and **5a**.

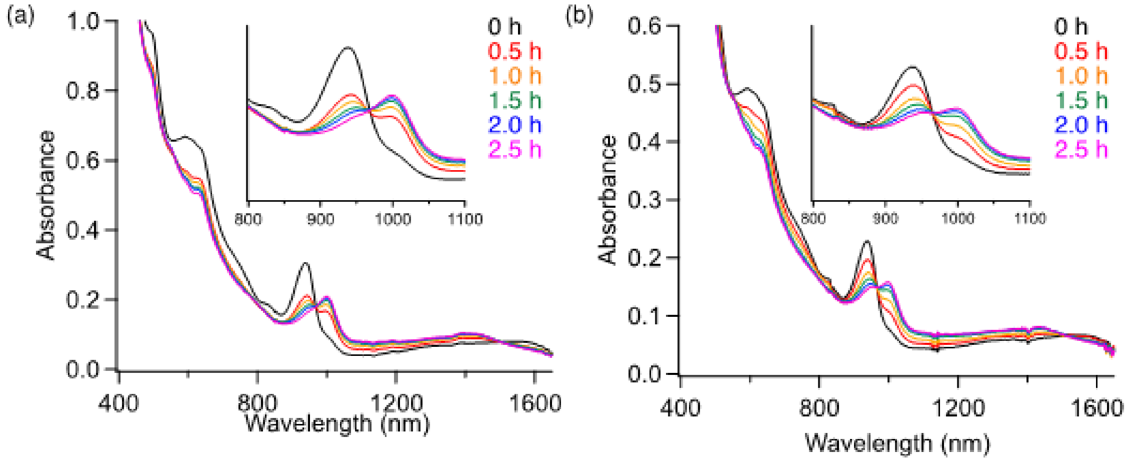


**Figure S2:** Changes in absorption spectra during the reaction of La@$C_{2v}$-$C_{82}$ with (a) **1b** and (b) **1c**.

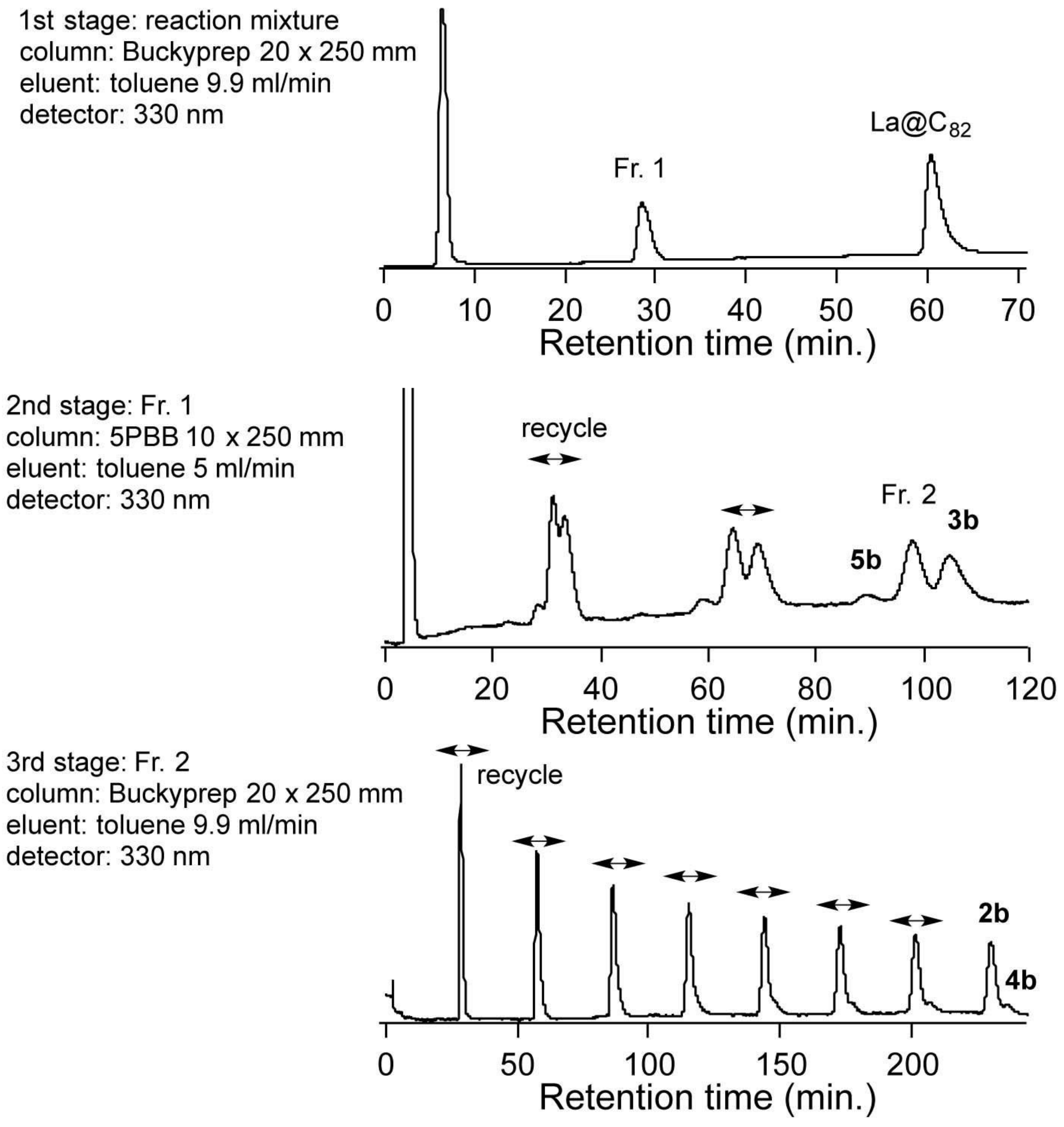


**Figure S3:** HPLC separation/isolation schemes for **2b**, **3b**, **4b**, and **5b**.

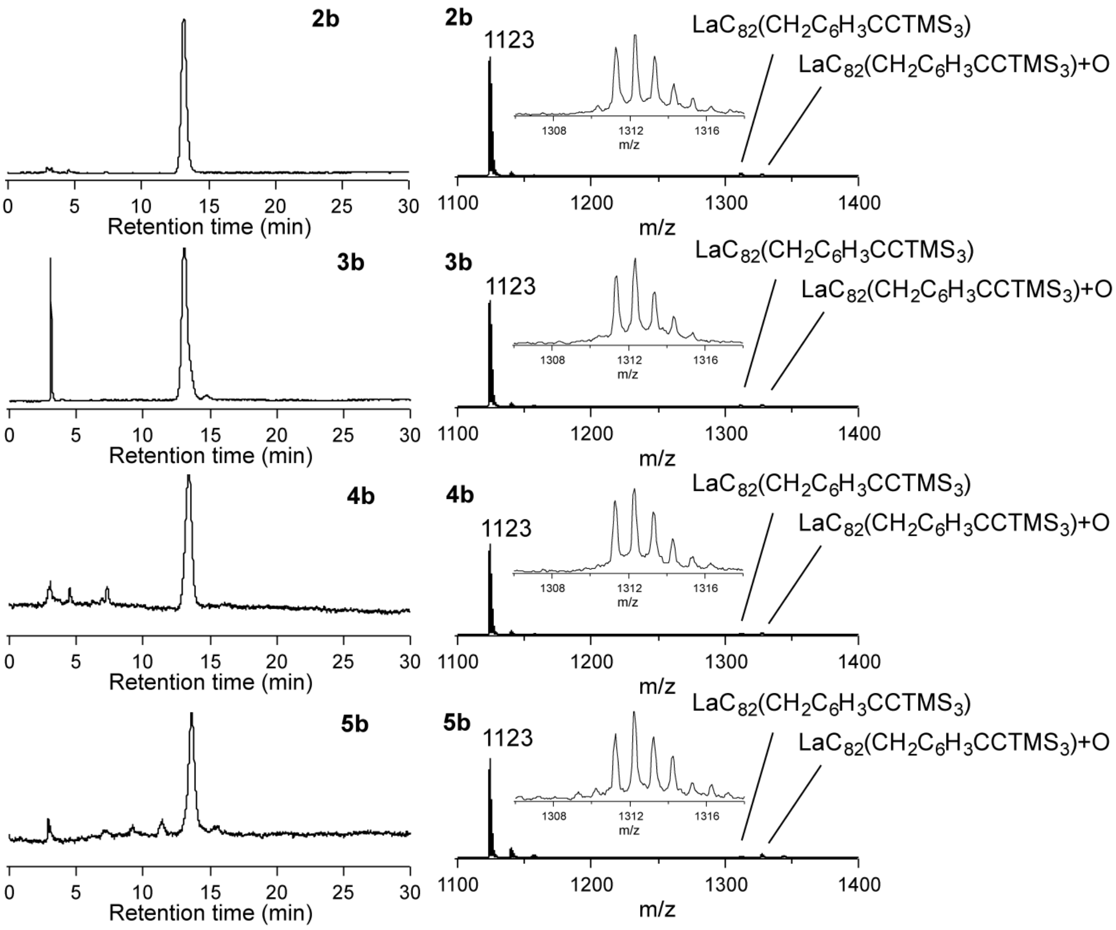


**Figure S4:** HPLC profiles and MALDI–TOF mass (positive mode) spectra of **2b**, **3b**, **4b**, and **5b**. HPLC conditions: column, Buckyprep (4.6 mm × 250 mm); eluent, toluene; flow rate, 1.0 mL/min; detector, UV detector, 330 nm.

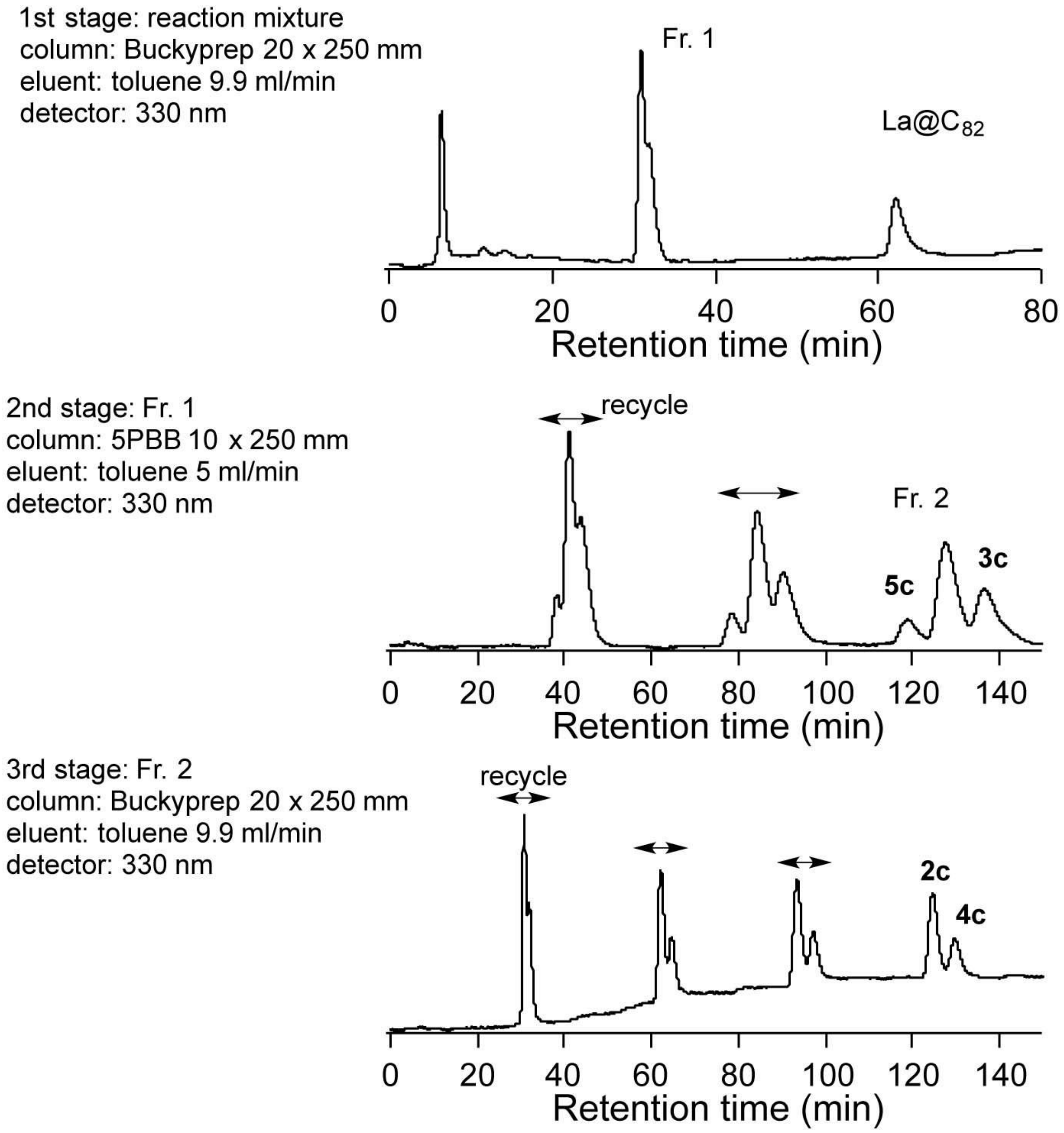


**Figure S5:** HPLC separation/isolation schemes for **2c**, **3c**, **4c**, and **5c**.

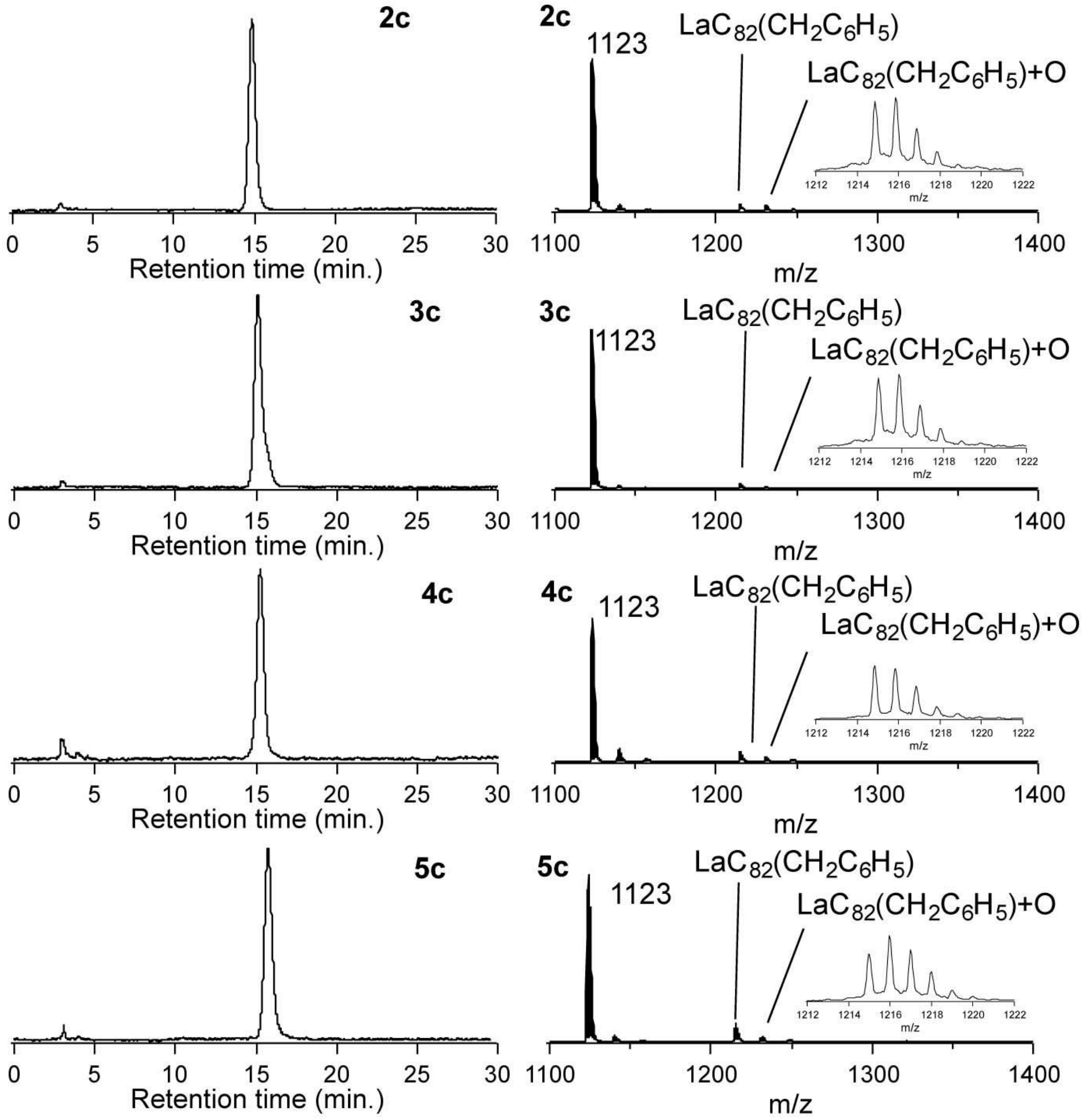


**Figure S6:** HPLC profiles and MALDI–TOF mass (positive mode) spectra of **2c**, **3c**, **4c**, and **5c**. HPLC conditions: column, Buckyprep (4.6 mm × 250 mm); eluent, toluene; flow rate, 1.0 mL/min; detector, UV detector, 330 nm.

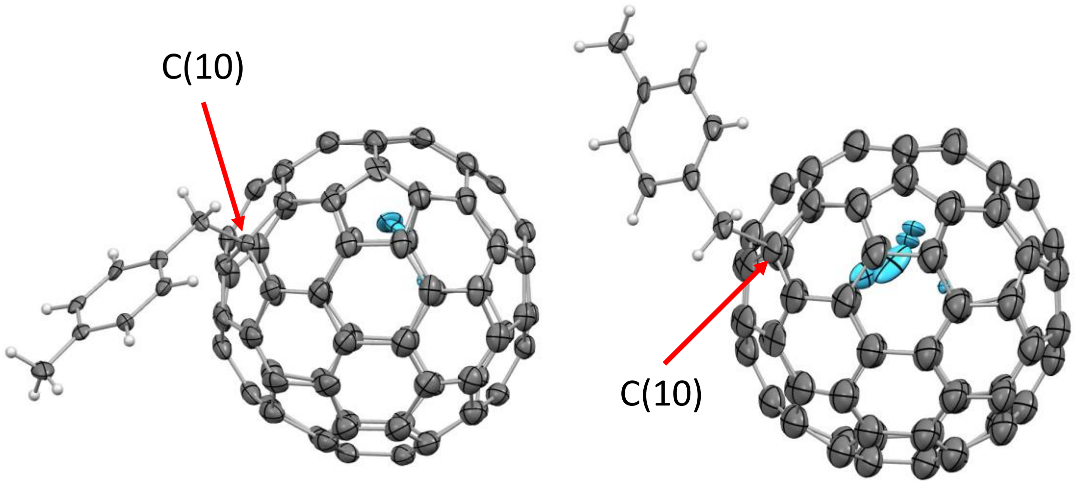


**Figure S7**: ORTEP drawings of an independent unit of **3a**.

**Table S1:** Crystal data of 2(**3a**)•$3CS_2$ (CCDC No. 2299232).

| | |
|---|---|
| formula | $C_{183}H_{18}La_2S_6$ |
| formula weight | 2686.16 |
| color, habit | black, block |
| crystal system | monoclinic |
| space group | $P2_1$ (No. 4) |
| $T$ (K) | 90 |
| $a$ (Å) | 16.4784(8) |
| $b$ (Å) | 14.5513(8) |
| $c$ (Å) | 19.6146(9) |
| $\alpha$ (º) | 90 |
| $\beta$ (º) | 90 |
| $\gamma$ (º) | 90 |
| $V$ (Å$^3$) | 4703.2(4) |
| $Z$ | 2 |
| $\rho_{calc}$ | 1.897 |
| $\mu$ (mm$^{-1}$) | 1.110 |
| crystal size (mm) | 0.690 x 0.240 x 0.180 |
| radiation ($\lambda$/Å) | fine-focus sealed tube (0.71073) |
| reflection collected | 70164 |
| independent reflections | 36557 |
| data ($I > 2\sigma(I)$)/parameter/restraints | 33932/1771/4430 |
| $R_{int}$ | 0.0486 |
| $R_1$/$wR_2$/GOF (all data) | 0.2235/0.5384/2.461 |
| $R_1$/$wR_2$/GOF ($I > 2\sigma(I)$) | 0.2180/0.5297/2.554 |

The reason for the large $R$ value is that the quality of the crystal is poor. This crystal data suggested an orthorhombic space group, but an initial structure could not be obtained. Therefore, the initial structure was shown in the monoclinic space group when the symmetry was lowered. This crystal data was analyzed based on this initial structure.
